# NK cell-derived extracellular vesicles enhance cytotoxicity and immune cell recruitment in non-small cell lung cancer

**DOI:** 10.3389/fimmu.2025.1633010

**Published:** 2025-07-24

**Authors:** Joanna Palade, Eric Alsop, Nanyun Tang, Jerry Antone, Dorothy M. Paredes, Tithi Ghosh Halder, Raffaella Soldi, Taylor Bargenquast, Gary Schwartz, Jennifer Finholt, George J. Snipes, Sunil Sharma, Michael Berens, Timothy G. Whitsett, Kendall Van Keuren-Jensen, Ronan J. Kelly

**Affiliations:** ^1^ Translational Genomics Research Institute (TGen), Phoenix, AZ, United States; ^2^ The Charles A. Sammons Cancer Center, Baylor University Medical Center, Dallas, TX, United States

**Keywords:** NSCLC, lung cancer, extracellular vesicles, NKEVs, CITE-seq, transcriptomics

## Abstract

**Introduction:**

Immune-based agents, especially Immune Checkpoint Inhibitors (ICI), are standard of care therapy in non-small cell lung cancers (NSCLC); however, a significant number of patient tumors fail to respond, or develop resistance. While target expression, mutation burden and oncogenic pathways impact responses, an established mechanism contributing to ICI therapy failure is evasion of T-cell responses via downregulation of human leukocyte antigen (HLA). Conversely, natural killer (NK) cells effector function is enhanced in the absence of HLA, making NK cellular therapies an attractive option for ICI resistant tumors. Challenges for current NK cell therapies include failure to adequately infiltrate solid tumors and long-term persistence, which may be overcome by deploying NK-derived extracellular vesicles (NKEVs) as a personalized novel adoptive cellular therapeutic with cytotoxic effects.

**Methods:**

In a human NSCLC cohort (n=10), we used single cell RNAseq and antibody labeling (CITEseq) to examine the immune cell landscape in peripheral immune cells (PBMCs) and tumors. NKEVs retrieved from patient NK cells were characterized with proteomics and bulk RNAseq, and EV functionality was assessed using primary tumor organoids.

**Results:**

We identified circulating NK cell subsets, describing differences in cell composition, gene expression and signaling, related to time point, NSCLC subtype (adenocarcinoma, squamous cell), composition and tumor grade. Next, we examined the functional capabilities of patient NKEVs in organoid structures derived from primary tumor cells, finding that exposure to patient NKEVs resulted in a 40-45% decrease in organoid viability, and significantly lowered the cisplatin dose required to elicit cytotoxicity. In Nivolumab treated PBMC co-culture experiments, NKEV addition favorably shifted the organoid infiltrating immune population to significantly fewer CD4+ T cells and more CD56+ NK cells. Finally, we used the multi-omic characterization of NKEV molecular cargo to identify RNA transcripts and proteins associated with cytotoxic and immune recruiting functions.

**Conclusions:**

This work demonstrated that NKEVs can be successfully harvested from patient derived, expanded NK cells, and highlights their heterogeneous cargo, and anti-tumor properties in combination with standard-of-care therapies.

## Introduction

Despite advancements in early detection, personalized medicine, and more recently, immunotherapy, 1.8 million people die globally of lung cancer every year, making it the most common cause of cancer-related death (1). Non-small cell lung cancer (NSCLC) accounts for 85% of cases, with lung adenocarcinoma (LUAD) and lung squamous cell carcinoma (LUSC) as the most common subtypes (2). Course of treatment is dictated by histology, tumor stage, medical history, molecular features (*e.g.*, driver mutation events and PD-L1 status. In particular, immune checkpoint inhibitors (ICIs) (especially PD-1/PD-L1 inhibitors and CTLA-4 inhibitors) have considerably improved outcomes, although a large subset of patients do not respond well, and recurrence rates remain high (3).

Response and resistance to ICIs are governed by various mechanisms, including PD-L1 expression, tumor mutation burden (TMB), mismatch repair pathways, microsatellite instability, oncogenic pathway expression, and the tumor microenvironment (4, 5). Dysregulation of human leukocyte antigen (HLA) expression and antigen presentation pathways in cancer cells contribute to ICI resistance and enable tumor evasion of T-cell responses (6). Numerous clinical trials have studied various combination partners of ICIs in NSCLC over the last decade but results have been disappointing, with limited advancements beyond targeting the PD-1/PD-L1/CTLA-4 axis (7). Unfortunately, the majority of NSCLC are immunologically “cold”, with a lack of activated tumor specific T cells in the immune microenvironment (TME) (8). Chimeric antigen receptor (CARs) and T cell receptor (TCRs) strategies can overcome the lack of T cells, but these cellular therapeutics have encountered challenges, including the absence of stable tumor antigen expression, impaired trafficking and the requirement for HLA restriction (9).

Conversely, natural killer (NK) cells are not limited by HLA mediated interactions to exert their functions, instead binding target cells via a repertoire of inhibitory and activating cell surface receptors (10). Thus, enhancing and leveraging NK cells’ ability to target tumor cells in the context of ICI therapy offers a promising solution, and is currently the topic of extensive investigation (11–15). While advances have been made, *ex vivo* expanded NK cells have limited persistence in circulation, and difficulty infiltrating solid tumors (16). Indeed, histological examination of lung tumor tissue finds NK cells in sparse numbers, exhibiting impaired cytotoxicity, and mostly occupying the periphery of the TME (17, 18). Additionally, NK cells are susceptible to soluble immunosuppressive molecules, most notably 1L-10, prostaglandin E2, macrophage migration inhibitory factor (MIF), indoleamine 2,3-dioxygenase, IL-37 and transforming growth factor-β (TGFβ) (19, 20). Hypoxic conditions within solid tumors, and failure to cross the blood-brain barrier and the blood-tumor barrier can also abrogate NK cell activity. To overcome these challenges whilst trying to preserve the benefits of NK cell therapeutics, it has been proposed that NK cell derived extracellular vesicles (EVs) may be utilized as a personalized novel next generation adoptive cellular therapeutic.

EVs are membrane encased, nanometer sized particles that carry molecular cargo derived from the cells of origin, with diverse functions such as cellular communication mediators, molecular waste disposal, and homeostasis maintenance (21, 22). Immune cell derived EVs play roles in antigen presentation, immunological synapse formation, and when released from effector cells, may target tumor cells (23–25). Experiments with EVs derived from primary and immortalized NK cells (NKEVs) have found the presence of miRNA cargo, cytotoxic enzymes (granzymes, perforin) and cytokines (IFNy, TNFa) that might lend NKEVs antitumor properties (26–29). While EVs retain many features of their parent cells, they also have characteristics that confer superiority as a cellular therapeutic over NK cells alone. Their protein composition can enhance cellular uptake and improve trafficking to target sites, while EV half-life, stability under physiological conditions, subcellular size, and abundance may further enhance tumor infiltration (30).

We used single cell transcriptomics coupled with surface antibody labeling on PBMCs and tumor infiltrating immune cells in NSCLC patients pre- and post-tumor resection and herein described differences associated with time-point, lung cancer subtype, and tumor grade, thus providing a view of the immune landscape, with a focus on NK cells. Additionally, we generated NKEVs from expanded NK cells and cataloged the repertoire of RNA and protein EV cargo contained within. Lastly, we interrogated the NKEVs’ cytolytic and immune recruiting capabilities in patient tumor cell organoid experiments, and integrated the transcriptomic findings, to explore EV cargo associated with anti-tumor properties.

## Methods

### Patient enrollment and sample collection

This study was approved by the institutional review board of Baylor Scott & White Health (022-032). All patients or their surrogates provided written informed consent for participation in this study. All samples (PBMCs and tumor tissue) were collected by trained medical staff. Whole blood was collected on the day of tumor resection surgery (samples designated as “pre”) and several weeks after surgery (“post”, 6.8± 7.1 weeks later) and processed according to established protocols. Briefly, PBMCs were retrieved from whole blood using SepMate tubes (Stemcell Technologies) and Ficoll-Paque density gradient separation, assessed for viability and concentration with trypan blue, and viability preserved in Recovery™ Cell Culture Freezing Medium (Gibco). PBMCs were stored in vapor phase liquid nitrogen. Lung tumor tissue was collected during surgery, and excess tissue not required for pathological examination was, when available, set aside for snRNAseq and tumor organoid experiments.

### Single cell RNA sequencing preparation of PBMC samples

The following antibodies were used for CITE-Seq (Biolegend, constant lot source was used across all experiments): TotalSeq™-C0083 anti-human CD16, barcode AAGTTCACTCTTTGC, TotalSeq™-C0084 anti-human CD56 (NCAM) Recombinant, barcode TTCGCCGCATTGAGT, and TotalSeq™-C0090 Mouse IgG1, κ isotype Ctrl, barcode GCCGGACGACATTAA. Antibody cocktail was prepared in protein lo-bind tubes, in flow staining buffer (2% FBS 2% BSA) and centrifuged 10 minutes 4C at 14,000xg, to pellet antibody aggregates. Freshly thawed PBMCs were resuspended at 1e6 cells/100 ul in flow staining buffer, and non-specific binding was blocked 10 minutes at 4C with TruStain FcX reagent. Antibody cocktail was added at a concentration of 0.5 ug/1e6 cells, and incubated 4C for 30 minutes. The stained cells were washed twice in flow staining buffer, to remove excess antibody.

Stained cells were filtered through a 70μm cell strainer, counted, and stained with NucBlue Live Ready Probes Reagent (ThermoFisher) and Calcein-AM (Invitrogen). Following cell counts, 15,000 cells were mixed with Chromium Next GEM Single Cell 5’ v2 GEM-RT master mix and loaded onto the 10x Genomics Chromium Next GEM Chip K. After purification of barcoded first strand cDNA, poly-adenlyated mRNA and DNA from protein Feature Barcode were amplified. Full-length cDNA was quantified using High Sensitivity D5000 screentape (Agilent Technologies). V(D)J and 5’ Gene Expression libraries were produced and pooled with amplified DNA from Feature Barcode TotalSeq libraries. Libraries and pools were quantified using High Sensitivity D1000 screentape (Agilent Technologies) and Kapa Library Quantification Kit for Illumina platforms. Equal volume library pools were quality controlled and rebalanced by shallow sequencing on iSeq 100 v2 flowcells (Illumina). Upon rebalancing by reads per cell, libraries were repooled, quantified by High Sensitivity D1000 and Kapa Library Quantification Kit and loaded on NovaSeq 6000 S4 or NovaSeq X Plus 10B flowcells (Illumina) for high depth sequencing at 26x10x10x90 cycles.

### scRNA-seq data analysis for the PBMC samples

Fastq files from NovaSeq flowcells were combined and analyzed together using cellranger multi (v. 7.1.0) to encompass both gene expression (GEX) and antibody capture (CSP) data for all cells into a single filtered feature matrix. Filtered feature matrices were loaded into python (v. 3.9.7) using muon (31). Muon anndata objects were subset to GEX data for quality filtering using scanpy (v 1.9.2). Cells were filtered to remove any cell with > 5% mitochondrial RNA, > 5% ribosomal RNA or > 0.1% hemoglobin genes. Cell expressing less than 200 genes were also removed. Doublets were then removed using the scrublets package (32). The quality filtered dataset was then normalized and logged using the scanpy normalize_total() and log1p() functions. Top 6000 highly variable genes were calculated using highly_variable_genes() with flavor=“cell_ranger” and batch_key=‘sample’. Data was batch corrected by sample using SCVI on the highly variable gene set (33). SCVI embeddings were then used to generate nearest neighbor graphs and UMAPs. SCVI embeddings were then used to generate nearest neighbor graphs and UMAPs. Cell type annotations were performed using celltypist with the Immune_All_low reference and majority_voting model (34). CSP data was then visually overlaid on top of the GEX UMAP using Muon. Reclustering of the NK cell cluster was done by subsetting to all cells labeled as NK cells by celltypist and rerunning SCVI, followed by a new nearest-neighbor calculation and new UMAP generation. Subclusters were generated using the Leiden algorithm with a resolution of 0.2. Marker genes which define the NK subclusters were determined using the rank_genes_groups() function in scanpy with the wilcoxon method. Differential gene expression between sample groups in each cell type was performed with the pseudobulk method muscat using the limma-voom strategy (35). scRNAseq data for the PBMC cohort is publicly available and may be accessed through GEO with accession number GSE274588.

### Single nucleus RNA sequencing preparation of tumor tissue samples

Since the tissue was flash-frozen and retrieval of intact single cells is not feasible, snRNAseq was used. Briefly, tumor tissue (~50mg) was homogenized in 1 ml of Nuclei EZ Lysis Buffer supplemented with 1x cOmplete Protease Inhibitor Cocktail (Sigma-Aldrich, NUC101-1KT and 4693116001, respectively) and RNasin Plus (Promega, N2611). The homogenate was passed through a 70 µm 1.5 ml mini strainer (PluriSelect) and centrifuged at 4°C, 500xg for 5 minutes. The nuclei pellet was resuspended in an additional 1 ml of Nuclei Lysis buffer and incubated for 5 minutes on ice followed by centrifugation at 4°C, 500xg for 5 minutes. 500 ul wash/resuspension buffer (2% BSA, 0.2 U/ul RNasin Plus in PBS) was added to the nuclei pellet and incubated for 5 minutes to allow adequate buffer exchange followed by centrifugation at 4°C, 500xg for 5 minutes, repeated once more, and finally the nuclei were resuspended in 500 ul wash/resuspension buffer. Resuspended nuclei were incubated with 1–2 drops of NucBlue Live ReadyProbes Reagent and immediately sorted using the DAPI channel on the Sony SH800S (Sony Biotechnology) with a 100 µm chip.

Nuclei were sorted for 15,000 events directly into 10x 5’ v2 RT Reagent Master Mix and immediately processed with the 10x Genomics Chromium Next GEM Single Cell 5’ v2 (Dual Index) kit (10x Genomics). Samples were loaded, cDNA amplified, and library constructed following the manufacturer’s protocol. As done for the PBMC scRNAseq libraries, multiplexed pools were analyzed with High Sensitivity D1000 screentapes, Kapa Library Quantification (Roche) and sequenced at shallow depths on Illumina’s iSeq 100 v2 flow cell for 26x10x10x90 cycles to determine estimated reads per cell. Upon rebalancing by reads per cell, libraries were re-pooled, re-quantified with the Kapa Library Quantification Kit and loaded on NovaSeq 6000 S4 or NovaSeq X Plus 10B flowcells (Illumina) for high depth sequencing at 26x10x10x90 cycles with 1% PhiX.

### snRNAseq analysis for tumor tissue samples

Analysis was carried out similarly to the PBMC workflow, with notable differences and quality filtering parameters appropriate for single nuclei sequencing. Briefly, cellranger count (v. 7.1.0) was used to capture gene expression (GEX) data for all nuclei into a single filtered feature matrix and filtering to remove nuclei with > 1% mitochondrial RNA, > 1.5% ribosomal RNA, > 0.1% hemoglobin genes, or <200 genes was done. Cell type annotations were performed using celltypist using the Human_PF_Lung reference and majority_voting model (34). snRNAseq data for the tumor tissue samples is publicly available and may be accessed through GEO with accession number GSE274595.

### NK cell isolation and culture

Frozen PBMC were thawed and spun in EasySep buffer (STEMCELL Technologies), followed by DNase treatment at 1 mg/ml in EasySep buffer for 10 minutes to encourage singularization. The cells were filtered through 40 um mesh strainers, and in cases where RBCs were visibly present (as a reddish or pink cell suspension), RBC lysis was carried out for 5 minutes at room temperature, with constant agitation, using eBioscience 1X RBC Lysis Buffer (ThermoFisher). The cells were washed in EasySep buffer, counted with Trypan blue, and NK retrieval was carried out according to the manufacturer’s instructions, with the negative selection based EasySep Human NK Cell Enrichment Kit (STEMCELL Technologies). Briefly, cells were resuspended at 5e7 cells/ml in EasySep buffer, and incubated for 10 minutes with Enrichment Cocktail, followed by a 5 minute incubation with Magnetic Particles. The bead-bound PBMC populations were magnetized for 3 minutes, the supernatant was retrieved, and the cells were washed, counted, resuspended in growth medium, and cultured at 37°C in a 5% CO2 humidified incubator. Growth medium was TheraPEAk X-Vivo 10 basal medium (Lonza), supplemented with 5% heat inactivated human male AB serum (Sigma Aldrich), 100 U/mL human IL-2 (Miltenyi) and 10 ng/mL human recombinant, animal free IL-15 (Biotechne). Cells were maintained at 1e6 cells/ml in growth medium for 12 days, with regular counting, cell splitting, and medium supplementation as needed, on days 5,7, and 10.

### NK cell culture for EV retrieval

To deplete medium derived EVs, the heat inactivated human male AB serum was treated with the FBS Exosome Depletion slurry kit (Norgen Biotek). Briefly, heat inactivated serum was supplemented with 20% basal medium, and ExoC buffer. The pH was measured, and ExoC buffer was added in small increments until the pH reached 8.5-9. After incubation with Slurry E, the EV free serum supernatant was retrieved and passed through a 0.2 um filter. On day 12, NK cells were retrieved and resuspended in growth medium at 0.6e6 cells/ml, and medium harversting was carried out every 48 hours, until days 18-24. Growth medium was TheraPEAk X-Vivo 10 basal medium (Lonza), supplemented with 5% EV depleted heat inactivated human male AB serum, 100 U/mL human IL-2, 10 ng/mL human IL-15, and 25 ng/ml human recombinant animal free IL-21 (Biotechne). The medium was spun first at 4C, 300xg 10 minutes, and again at 4C 2000xg 10 minutes to pellet additional debris, followed by storage at -80C until EV isolation. No additional high-speed centrifugation was carried out, thus preserving the large vesicle population within the CCM milieu (apoptotic bodies, microvesicles).

### EV isolation and characterization

Cell culture medium (CCM) was thawed and concentrated 10-fold with Amicon 10kD cutoff filters (Milipore). EVs were retrieved using size exclusion chromatography (SEC) with Gen 2–70 nm resin columns spanning the qEV1–10 size, depending on CCM volume (Izon). Briefly, columns were equilibrated with filtered and degassed PBS, followed by concentrated CCM application. Once the CCM entered the resin, the column was topped off with additional degassed PBS, and the eluate was collected up to fraction 10–11 and concentrated again via Amicon 10kD filters. Concentrated EVs were then aliquoted to minimize freeze-thaw cycles, and particle size and distribution was examined using nanotracking analysis (NTA) on a NanoSight300 instrument (Malvern Analyticals), using 3 captures for 30 seconds each per sample. The NTA data was analyzed using NTA 3.4 software. To determine protein concentration, intact EVs were analyzed with a microBCA assay and a 0.0025- 0.2 mg/ml standard curve.

### NKEV and cell culture medium immunoblotting

The Proteome Profiler Human Cytokine Array Kit (R&D Systems) was used according to the manufacturer’s instructions. 500 ul CCM was lysed in RIPA buffer supplemented with Halt Protease Inhibitor (Thermo Scientific) for 30 minutes on ice, with intermittent vortexing. Samples were incubated with antibody cocktail 1 hour with constant agitation, and applied to the blocked membranes overnight at 4°C. Washes and SA-HRP treatment were carried out according to the user protocol, and imaging was done on Odyssey’s LI-COR instrument. Semi-quantitative densitometry analysis was done with FIJI software, using the value of the area under curve (AUC). Data were normalized to the positive control average. For immunoblotting, the following primary antibodies were used: mouse monoclonal anti-human Perforin (R&D systems, MAB8011, 1:20 dilution), mouse monoclonal anti-human Granzyme B, (R&D systems, MAB2906, 1:30 dilution), rabbit polyclonal anti-human Alix (Novus Biologicals, NBP1-49701, 1:20), rabbit monoclonal anti-human Annexin A2 (Cell Signaling Technology, 8235, 1:80). NKEVs were lysed with RIPA for 30 minutes on ice, total protein was quantified via microBCA assay, and Western blots were carried out on a Jess ProteinSimple instrument per manufacturer’s instructions, as follows: EV protein lysates were diluted to 0.99 mg/ml, denatured at 95C for 7 minutes with DTT buffer and fluorescent standards, and loaded onto 12–230 kDa Jess plates. Ready-to-use HRP-conjugated anti-rabbit and anti-mouse secondary antibodies (R&D) were used, and the instrument was run with a 25-capillary cartridge, using standard settings for chemiluminescence detection. For proteomics analysis, aliquots of EVs were submitted for processing and analysis to the TGen Collaborative Center for Translational Mass Spectrometry core.

### Whole transcriptome library preparation and sequencing

NKEVs isolated with SEC were prepared for RNAseq as follows: RNA was isolated with Norgen Biotek’s Plasma/Serum Circulating and Exosomal RNA Purification Mini Kit (51000), according to the manufacturer’s instructions. Samples were twice incubated in lysis buffer with 2-mercaptoethanol and slurry at 60°C, and nucleic acids were precipitated with ethanol, captured onto the column, washed, and eluted with RNase free DNase free ultra-pure water. DNAse treatment was done with TURBO DNA-free (ThermoFisher) at 37C for 30 min, and following DNase inactivation, the RNA was cleaned and concentrated with RNeasy MinElute Cleanup (Qiagen) according to the manufacturer’s instructions.

RNA concentration was assessed via Quant-iT RiboGreen (ThermoFisher), and up to 15 ng RNA were used for library preparation with SMARTer Stranded Total RNA-Seq Kit v3 - Pico Input Mammalian and SMARTer RNA Unique Dual Index Kits (Takara Bio) Strand-specific, dual-indexed libraries were generated with the following parameters: RNA fragmentation 94C for 2 minutes, 5 cycles PCR1 and 13 cycles PCR2. Libraries were quantified using KAPA SYBR FAST Universal qPCR Kit (Roche) and Agilent’s TapeStation 4200 instrument with the D1000 High Sensitivity kit, and combined into an equimolar pool spiked with 1% PhiX. Sequencing was carried out on Illumina’s NovaSeq 6000, on one lane of a 200 cycle S1 flow cell with XP workflow, to 50 million paired end reads.

### NKEV whole transcriptome RNAseq data analysis

Fastq files from whole transcriptome data were combined and then aligned using STAR v2.6+ (36) to GRCh38 and to additional lncRNAs from the highly curated LNCpedia (37) and GENCODE datasets. Count data were curated using featureCounts (38). Differential gene expression analysis was performed in R (v.4.2.2) using featureCounts output tables. Count tables were filtered to include only genes present at >1 count in >50% of samples, and DEG analysis was performed using DESeq2 (v. 1.38.5) using filtered counts tables. Whole transcriptome EV RNAseq data is publicly available and may be accessed through GEO with accession number GSE274584.

### Fresh tumor dissociation and preparation

Following tumor excision after surgery, the tissue was placed in 10 ml of MACS Tissue Storage Solution (Miltenyi) at BSW and shipped overnight at 4C to TGen, where it was received and immediately processed using the Tumor Dissociation Kit (Miltenyi), according to manufacturer’s instructions. The tissue was rinsed with PBS, weighed, and cut into 2 mm pieces with sterile forceps. The minced pieces were placed in a gentleMACS C tube, in DMEM/F12 (Invitrogen) medium supplemented with enzymes H, R and A and incubated in a gentleMACS Dissociator with the 37C_h_TDK_2 program. After enzymatic digestion, the tissue was centrifuged, resuspended, and passed through the 70 um MACS SmartStrainer to retrieve single cells. The dissociated lung tumor cells were counted, resuspended in freezing medium (90% FBS 10% DMSO) until ready for the organoid experiments.

### HCC827 cell culture

Lung cancer cell line HCC827 was maintained in RPMI-1640 medium (ThermoFisher, 11875093) supplemented with 10% FBS, and 1x pen/strep with standard culture conditions, for organoid generation experiments.

### Organoid NKEV and cisplatin treatment

Dissociated patient tumor cells were resuspended in DMEM/F12 medium supplemented with 20 ng/ml of bFGF (Invitrogen), 50 ng/ml of human EGF (Invitrogen), 1% N2 (Gibco) and 2% B27 (Gibco). 1000 cells were seeded into non-adherent 384 well u- bottom plates (S-Bio) in 25ul of medium with 2% Matrigel (Sigma-Aldrich) per well. The 384 plates were spun at 1000 RPM for 2 minutes, and cells were grown at 37°C in a 5% CO2 humidified incubator, to allow organoid formation. After 6 days, a dose dependent response of cisplatin (Sigma-Aldrich) was then tested on the organoids with a 7-point, 1:2 serial dilution starting with an initial concentration of 100 μM. Organoids were treated with a single dose of 10 ug NKEVs derived from a pool of healthy donors, or expanded patient NK cells, both alone or in combination with cisplatin at 6.25 uM, 12.5 uM and 25 uM. After 6 days post EV and cisplatin treatment, cell viability was measured with CellTiter Glo^®^ (Promega G9682). Experiments were carried out in 6 replicates per condition tested. Data was collected, normalized to its no-treatment vehicle control, and analyzed using GraphPad Prism 10.0.

### Organoid PBMC and NKEV transwell experiments

Dissociated patient tumor cells were resuspended in phenol free DMEM/F12 based medium (Invitrogen) medium supplemented with 10% FBS (R&D), 1x non-essential amino acids (Gibco), 1 mM Sodium Pyruvate (Gibco), pen-strep (Gibco) and Normocin (Invivogen). In black-rimmed, non-adherent clear u-bottom 96 well plates (Corning), 5,000 cells per well were seeded in 40 ul complete medium with 1.5% Matrigel, and centrifuged for 2 minutes at 2000 RPM, followed by culture at 37°C in a 5% CO2 humidified incubator. After 3 days, an additional 60 ul complete medium/well was added, and transwell assays were carried out at day 6 post-seeding. PBMCs were thawed 24 hours prior to the assay, counted with Trypan Blue, and maintained in CTS OpTimizer T-cell Expansion Medium prepared according to manufacturer’s instructions, (ThermoFisher) at 1e6 cells/ml in ultra-low attachment cultureware (Corning). The day of the assay, Nivolumab antibody (Sellekt chem) was added to the PBMCs at 10 ug/ml, and cells were incubated with antibody at 37C for 45 minutes with constant agitation. Excess antibody was removed with 2 washes in EasySep buffer, and the PBMCs were resuspended in CTS OpTimizer medium supplemented with 2 uM Molecular Probes Vybrant CM-Dil (ThermoFisher), at 1e6 cells/ml. PBMCs were incubated with DiI for 45 minutes at 37C, and 15 minutes at 4C, then washed twice again, and resuspended in phenol free organoid medium. During the PBMC treatment, the day 6 organoids are treated with 20ug NKEVs, 6.25 uM cisplatin, or both, and supplemented with medium up to 200 ul. The 96 well organoid plate was fitted with a 5 um pore size HTS permeable transwell plate (Corning), and PBMCs were added to the top of the transwell at 50,000 cells in a 100 ul volume. The co-culture assays were incubated at 37C for 48 hours, and PBMC infiltration was measured via Z-stack imaging and flow cytometry. Briefly, transwell inserts were removed without disturbing the organoids, and 3D Z-stack imaging and morphometric analysis on a Cytation 5 instrument (Agilent) was used to measure the extent of lymphocyte infiltration using the RFP 531,593 channel. Experiments were carried out in 3–6 replicates, depending on EV and organoid availability. To ensure enough cells for flow cytometry, organoids from 3–6 experiments were pooled, washed twice in PBS to remove non-infiltrating PBMCs, and incubated with 1x Trypsin EDTA at 37C for 5–20 min to dissociate the organoids. The singularized cells were washed, resuspended in flow staining buffer containing Zombie Violet viability stain, and non-specific blocking was done with TruStain FcX block for 10 min at room temperature. Cells were stained with antibodies against CD4-FITC, CD8-APC and CD56-BV605 (Biolegend, 5 ul/1 million cells), for 30 minutes at 4C, followed by 2 washes and resuspension in flow staining buffer. Flow cytometry experiments were carried out on a CellStream instrument (Luminex) and analyzed using CellStream Analysis software. Briefly, the instrument was calibrated before each run, and compensation was carried out with single labeled PBMCs. Zombie Violet positive cells were excluded, and DiI staining was used to identify labeled PBMCs. Percentages of CD4+, CD8+ and CD56+ cells were calculated from the live, DiI gated cells.

## Results

### Transcriptomic landscape of circulating and tumor infiltrating immune cells

**Figure 1:**
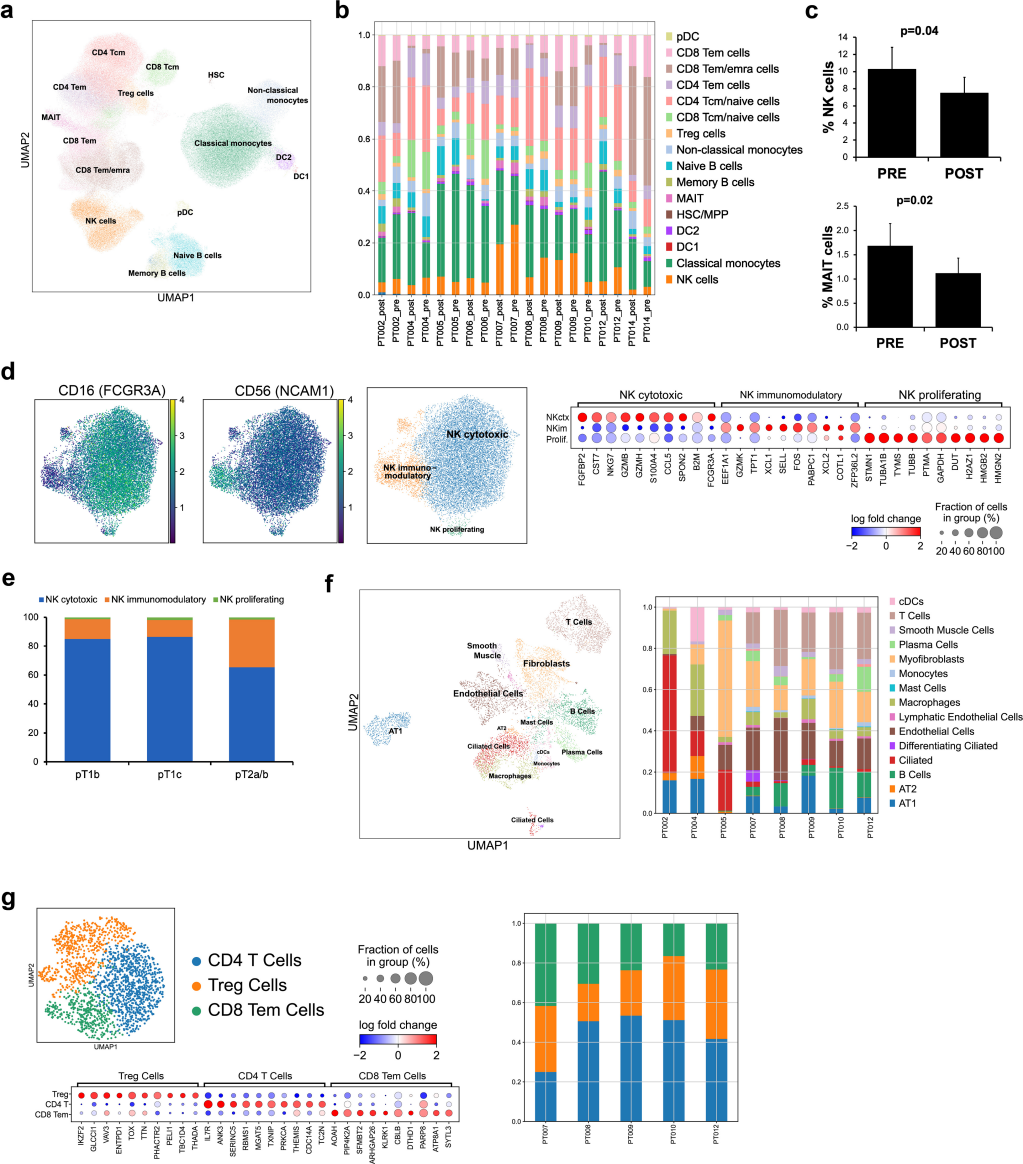
Profiling circulating and tumor infiltrating immune cells in a cohort of 10 NSCLC patients. scRNAseq was used to profile PBMCs pre and post tumor resection from LUAD (7 patients; n=7 “pre”, n=6 “post”) and LUSC (3 patients, n=3 “pre”, n=3 “post”) samples and whole tumor tissue retrieved following resection (n=8) was analyzed with snRNAseq. The overall distribution of PBMCs in UMAP form **(a)** and separated by sample **(b)** showed T cells as the most abundant cell type identified, present in various naïve/effector and helper/cytotoxic subsets, followed by monocytes. The proportion of NK and MAIT cells decreased post resection **(c)**. CITE-seq with cell surface antibody labeling of PBMCs showed localized expression of CD56 and CD16 to specific subclusters marking cytotoxic, immunomodulatory, and proliferating NK cells (n=10 “pre”, n=9 “post”) **(d)**. Examining the ratio of NK subtypes in patients with different grade tumors (pT1b, n=12, pT1c, n=4, pT2a/b, n=4) found a significant shift to a larger number of NKim in higher grade tumors **(e)**. The overall distribution of identified cells at the tumor site shown as a UMAP and separated by sample shows a heterogeneous mixture of immune cells (T cells, B cells), vasculature (endothelial, lymphatic cells), and specialized resident lung cells (AT1,2, fibroblasts) **(f)**. Subclustering the tumor infiltrating T cell population and examination of the enriched transcripts in each subpopulation found 3 T cell types, present uniformly in 5 participants **(g)**. Error bars in **(c)** represent SEM and statistical analysis was done via t-test.

Our NSCLC cohort was composed of patients with LUAD (n=7) and LUSC (n=3) diagnoses, with an average age at sample collection of 64.5 ± 9.7 years old, both with a history of smoking (n=8), and without (n=2). Known genetic alterations (derived from the Endeavor next generation sequencing assay, which includes 500+ well-characterized relevant cancer genes with full exon coverage) are presented in **Table 1** and **Supplementary Table 1**. Viable PBMCs collected from participants pre- and post-tumor resection (post; 6.8± 7.1 weeks later) were processed for single cell RNAseq and a total of 183,810 cells passed filtering metrics (**Figures 1a, b**; **Supplementary Figures 1a, b**). Celltypist, which uses a logistical regression model based on published datasets, was used to assign cell identity. While samples from all patients and time points were represented in each PBMC cluster, there was considerable interindividual variability in cell distributions, and following surgery, a small but significant decrease in the NK and mucosal associated invariant T (MAIT) cell populations was noted (**Figure 1c**). LUAD and LUSC have different developmental origins, and common driver mutations (39). Differential gene expression analysis between LUAD and LUSC PBMCs found significant upregulation of *ERAP2*, which encodes an enzyme involved in antigen processing and presentation, in LUAD PBMCs, including NK cells, and significant upregulation of *CX3CR1*, the fractalkine receptor, whose expression may be associated with better therapy response in NSCLC, in the LUSC T and NK cells (**Supplementary File 2**) (39, 40).

**Table 1:** Cohort overview.

Patient ID	PT002	PT004	PT005	PT006	PT007	PT008	PT009	PT010	PT012	PT014
Age	60	51	59	64	75	57	65	57	80	77
Sex	F	F	M	M	F	M	F	F	F	M
Ethnicity	Hispanic	Black	White	White	White	White	White	White	White	Black
Smoking status	No	No	Yes	Yes	Yes	Yes	Yes	Yes	Yes	Yes
Tumor stage	pT1bN0 - stage 1	pT1bN0 - stage 1	pT2bN0 - stage IIA	pT1cN1 - stage IIB	pT1bN0 - stage 1	pT1bN0 - stage 1	pT1bN0 - stage 1	pT1bN0 - stage 1	pT1cN0 - stage 1	pT2aN0 - stage 1B
Diagnosis	Adeno- carcinoma	Adeno-carcinoma	Squamous CC	Squamous CC	Adeno-carcinoma	Adeno-carcinoma	Adeno-carcinoma	Adeno-carcinoma	Squamous CC	Adeno-carcinoma
TMB	5.4 muts/Mb	not available	0.0 Muts/Mb	9.2 Muts/Mb	23.8 Muts/Mb	9.2 Muts/Mb	4.6 Muts/Mb	not available	5.4 Muts/Mb	10.8 Muts/Mb
PD-L1 (22C3)	TPS 1-5%	TPS 5-15%	TPS 1-25%	TPS 1-49%	TPS <1%	TPS 10-25%	TPS 1-5%	TPS <1%	TPS 76-100%	TPS 1-5%
Genomic alterations	**KRAS**, G12D	None detected	ARID1A, PIK3CA, FBXW7	**EGFR,** FGFR1, CDKN2A, PIK3CA, MDM2	NF1, CCND1	None detected	**EGFR**	**KRAS**	BRIP1, CD274, PIK3CA, FBXW7	**EGFR**

NSCLC patient demographics are summarized, including cancer subtype and staging based on pathological examination. Tumor mutational burden and genomic alterations, as identified by the Endeavor sequencing assay, are also displayed.

Peripheral NK cells are divided into subtypes with distinct functions and cell surface receptors. We used CITE-seq and barcoded antibodies to quantitatively mark expression of CD16 and CD56 in circulating PBMCs (41). Based on gene expression and CITE-seq analysis, NK cells could be further divided into: immunomodulatory, cytokine secreting cells (CD56++ CD16-, *SELL, XCL1, XCL2*), cytotoxic, effector cells (CD56+ CD16+, *GZMB, GZMH, PRF1, FGFBP2*), and a small, intermediate proliferating cluster (CD56++ CD16+, *HMGB2, TUBA1B, MKI67*) (**Figure 1d**; **Supplementary Figures 1c, d**). As expected, most were cytotoxic (NK_ctx_, 81.2%), whereas proliferating cells were sparse (1.5%). The immunomodulatory subset (NK_im_), which generally makes up less than 10% of circulating NK cells, was elevated in our NSCLC patients (17.4± 9.4%). Specifically, in patients with higher grade tumors (n= 2), the ratio of NK_ctx_/NK_im_ cells was significantly altered, compared to patients with lower grade tumors (n= 8) (**Figure 1e**). Interestingly, in the NK_im_ subset, expression of *IL15* and inhibitory receptor *KLRG1* increased significantly with tumor progression; chemokine receptor *CXCR3* expression increased with tumor grade in both NK_ctx_ and NK_im_ cells (**Supplementary Figures 1e, f**).

Single nucleus RNAseq (snRNAseq) was successfully carried out on 8 lung tumor tissue samples from the same cohort; to focus on the immune signature, the clusters of cells belonging to the tumor were filtered out (**Figure 1f**; **Supplementary Figures 1g, h**). At the tumor site, both specialized (AT1, AT2, ciliated epithelial cells, lung fibroblasts), and vasculature related cells (endothelial, smooth muscle cells, lymphatic) were identified, reflecting the structural lung composition. Among the most abundant immune cell types found in the TME were T cells (13.9%), and macrophages (9.8%), followed by B cells and plasma cells (6.7% and 3.6%, respectively), and finally, dendritic cells (3.8%). There was marked sample-to-sample variation in cell distributions, likely due to the heterogeneous nature of the tumor composition and location (**Figure 1f**). Further examination of the T cell cluster alone found 3 distinct cell types at the TME, each with a similar distribution in the 5 patients with robust T cell infiltration (**Figure 1g**). Almost 80% of the cluster were CD4 naive/central memory (*IL7R*) and Treg cells (*IKZF2/Helios, TOX*), with the remaining subcluster allocated to effector CD8 T cells (*KLRK1, SYTL3*). No NK cells were definitively identified at the tumor site in our cohort.

Together, these analyses point to differences between circulating NK cells related to tumor subtype and grade, and found differential gene expression in antigen processing and presentation and chemokine signaling. At the TME, no NK cell clusters were identified, and cytotoxic T cells were sparse. Additionally, incorporating CITE-seq facilitated the examination of circulating NK cell states, and found a shift in subtype ratio associated with tumor grade.

### Expanded patient NK cells produce NKEVs with extensive RNA and protein cargo

**Figure 2:**
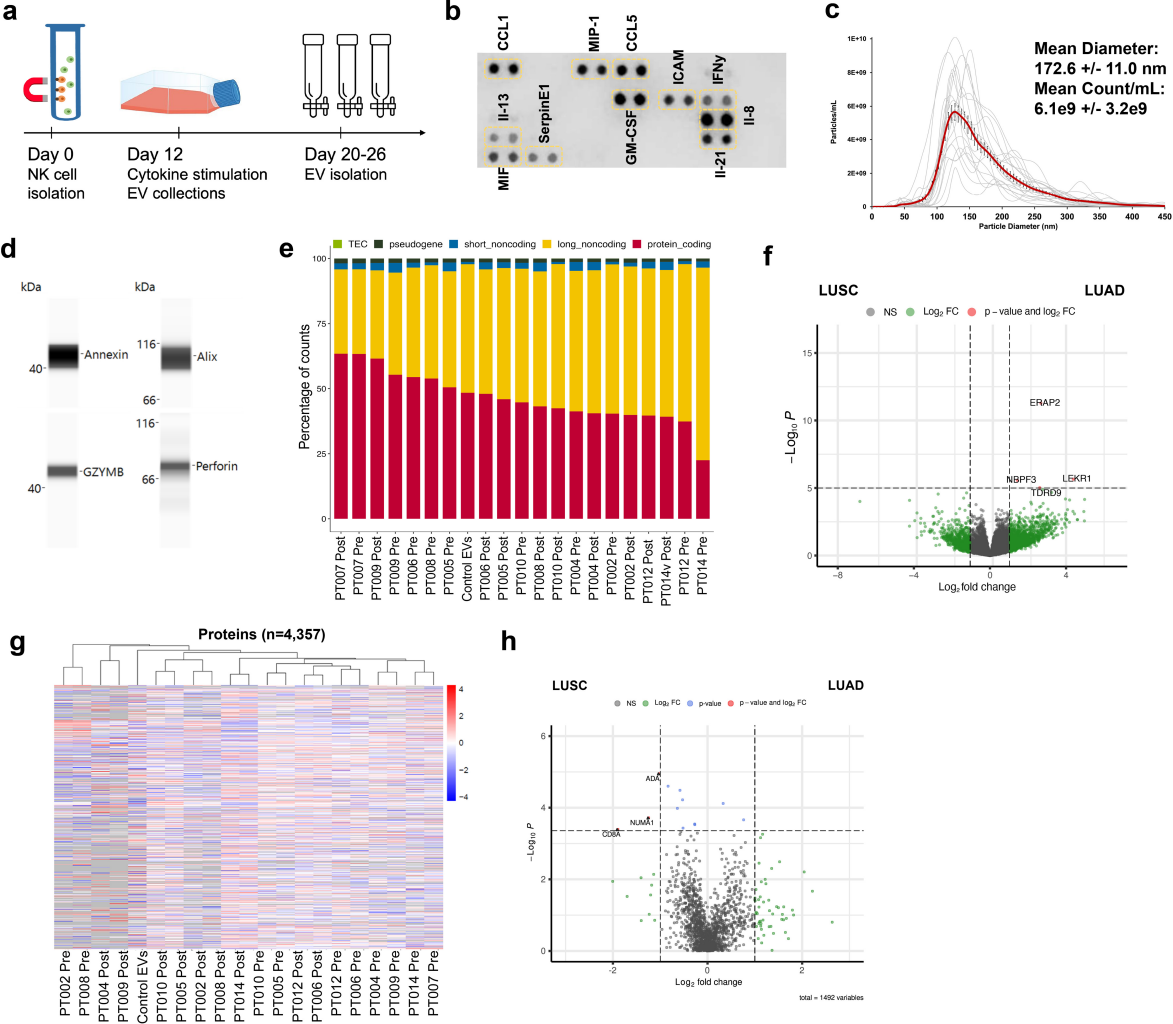
Patient derived NK cells can be expanded *in vitro* and NKEVs contain protein and nucleic acid cargo reflecting the cells of origin. NK cells were isolated from patient PBMCs via negative selection and EVs were isolated with size exclusion chromatography from the NK cell culture medium (n=20, 10 “pre” and 10 “post) **(a)**. Representative image of cytokine profiling (n=12) of the secretome showed the presence of classic chemokines (CCL1, -5) and cytokines (IFNy, GM-CSF) **(b)**. NKEVs were 100–200 nm in diameter post SEC (n=20) **(c)** and express canonical EV markers and cytotoxic NK proteins **(d)**. Whole transcriptome sequencing (n=21; 20 patient NKEV and 1 control pool NKEV) found the majority of NKEV RNA cargo is protein coding and long noncoding transcripts **(e)**. Differential gene expression (DE) analysis of LUAD/LUSC NKEVs identified a small number of significantly DE transcripts between LUAD and LUSC, such as ERAP2, which is upregulated in LUAD NKEVs **(f)**. Mass spectrometry proteomics analysis (n=21; 20 patient NKEV and 1 control pool NKEV) of NKEV cargo identified over 4000 proteins **(g)**, and modestly differentially expressed proteins between LUAD and LUSC groups **(h)**. **(b)** is one representative dot blot, PT007-post and **(d)** is one representative Western blot, PT007-post. **(c)** shows NTA analysis for all individual samples in grey, with the mean distribution in red and SEM error bars.

RNAseq analysis found sparse tumor infiltration of cytotoxic effector cells, underscoring the challenges of NK cell-based therapeutics. NKEVs could serve as a complementary treatment, both directly targeting tumor cells and potentiating the immune response. To explore the utility of patient derived NKEVs as an adoptive therapeutic, we isolated and expanded NK cells from patient PBMCs, and stimulated with IL-21 prior to EV retrieval and characterization (**Figure 2a**). Expanded NK cells expressed CD56 and CD16 pre and post stimulation (**Supplementary File 3**). Cytokine profiling of the cell culture medium showed NKs released proteins associated with recruitment, activation, and cytotoxicity (CCL5, GM-CSF, IFN-y, MIF) (**Figure 2b**, **Supplementary File 3**). NKEVs were isolated via SEC and ranged in size from 100–250 nm, with 4–7 billion particles/mL medium, and no significant size or concentration differences between pre/post tumor resection or LUAD/LUSC (**Figure 2c**; **Supplementary File 3**). Moreover, immunoblot analysis confirmed the presence of EV specific markers (Annexin A5, ALIX) and cytotoxic NK proteins (GZMB, Perforin) (**Figure 2d**). The RNA EV cargo was profiled using whole transcriptome RNAseq, and over 10,000 protein-coding (mRNA) and 2,000 long noncoding transcripts (lncRNA) were identified (counts >10) (**Figure 2e**). Protein coding genes associated with NK cell function (*GZMB, GNLY, NKG7, IL2RB, PRF1*) were among the most abundant, in agreement with the cytokine profiling and EV Western blot analysis (**Supplementary File 4**). A differential expression (DE) analysis (with sex and age as covariates; p adj.<0.05) found no significantly different genes when comparing pre- and post-samples (not shown) while tumor subtype comparisons found significant transcripts upregulated in LUAD EVs, like *ERAP2*, mirroring the NK cell transcriptomic signature (**Figure 2f**). Using mass spectrometry, around 4,000 proteins were detected, including classic cytotoxic proteins (GZMB/A/H/K, PRF1), NK cell receptors (NCAM1, CD244, KIR2DL1/2/3, KLRB/C/D, LAIR1, FCGR3A), and cytokines (IL-2, -10, -15, CCL1/5, CXCR3/4/6, TNF, INF-y) (**Figure 2g**). DE analysis between LUAD and LUSC NKEVs found several significant proteins (p adj. <0.05; **Figure 2h**; **Supplementary File 5**). Together, these data showed that patient PBMCs can be exploited for NKEV collection, and that, as expected, NKEV molecular cargo reflected NK cell identity and function.

### Patient derived NKEVs have cytotoxic properties

**Figure 3:**
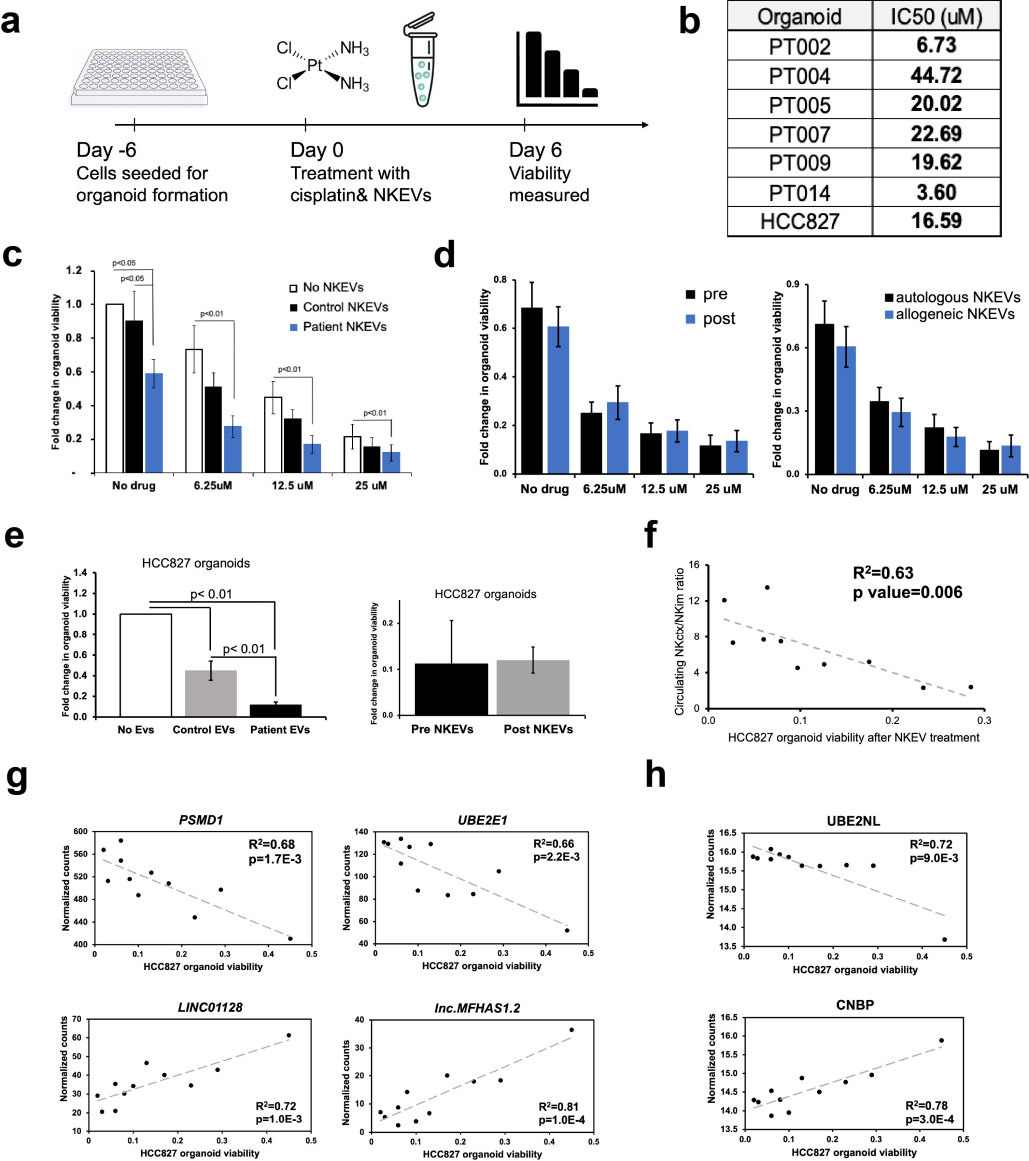
Patient NKEVs have potent cytotoxic abilities in tumor organoid experiments. NKEVs were directly added to lung cancer cell organoid structures, either alone or with increasing concentrations of cisplatin and viability was assayed via ATP production **(a)**. Cisplatin IC50 calculations for the cell line and patient derived organoids (with no NKEV treatment) ranged from 4–45 uM **(b)**. Treatment with either control NKEVs (derived from a pool of donors with no diagnosed cancer) and patient NKEVs results in a drop in organoid viability, both alone and in combination with increasing doses of cisplatin **(c)**. There are no significant differences in organoid viability when comparing pre and post NKEVs or when using autologous (NKEVs derived from the same patient as the organoid) and allogeneic (cross testing with NKEVs from a different patient) samples **(d)**. NKEV experiments with lung cancer cell line HCC827, in the presence of 25 uM cisplatin, showed HCC827 organoids were susceptible to patient NKEV treatment, and there were no significant differences between pre and post resection NKEVs **(e)**. There is a significant positive correlation between the starting ratio of cytotoxic to immunomodulatory NK cells, and the anti-tumor ability of the NKEVs derived from those cells (R^2^ = 0.63, p= 0.006) **(f)**. Representative RNA transcripts **(g)** and proteins **(h)** that correlate significantly with cytotoxicity are displayed as scatter plots with corresponding R^2^ and p values. Error bars in **(c)**, **(d)**, and **(e)** are SEM. Statistical analysis was done via one-way Anova with a Tukey HSD test **(c)**,**(e)** and t tests **(d)**. Organoid experiments were done in 6 replicates per condition.

EVs can modulate target cell activity via horizontal transfer of molecular cargo, and NKEVs have been shown to target cancer cells (26, 42). To test the NKEVs’ antitumor properties, NSCLC organoid structures were generated, when available, from singularized primary lung tumor resected during patient surgery, treated with cisplatin and/or NKEVs, and assessed for viability (**Figure 3a**; **Supplementary File 6**). Cisplatin IC50 values varied considerably among patient organoids (3.6- 44.7 uM), possibly reflecting the molecular heterogeneity present in NSCLC (**Figure 3b**). Treating the tumor organoids with 10 ug of patient-derived NKEVs alone, or in concert with increasing doses of cisplatin resulted in significant reduction in organoid viability, particularly at low/no cisplatin doses, where NKEV treatment affected viability by as much as 40-45%. Treatment with the same dosage of control NKEVs (derived from a pool of control NK cell donors with no cancer diagnoses) had only a slight and non-significant effect, potentially suggesting cancer patient NKEVs are uniquely cytotoxic (**Figure 3c**). There was no significant difference between treatment with NKEVs from cells pre- or post-tumor resection, or autologous vs allogeneic NKEVs (*i.e.*, autologous- both organoid and NKEVs derived from the same patient; allogeneic- NKEV source is a different patient) (**Figure 3d**). Certain NKEV-organoid combinations had a greater effect than others, although it is difficult to discern whether that is due to EV functionality or patient organoid susceptibility to NKEVs. To address this, experiments were also carried out with uniform organoids derived from established lung cancer cell line HCC827 (LUAD, EGFR mutant), treated with cisplatin and patient NKEVs. As noted with the patient experiments, there was no significant difference between treatment with -pre or -post NKEVs (**Figure 3e**). Interestingly, examining the average NKEV cytotoxicity and the starting distribution of NK subsets (as determined from the scRNAseq data) yielded a significant correlation between organoid viability after NKEV treatment and starting ratio of cell subtypes, where EVs derived from NK cells with a larger population of NK_ctx_ had greater anti-tumor effects (**Figure 3f**). To determine whether the molecular cargo was associated with cytotoxicity, we performed linear regression analysis between the viability loss in HCC827 organoids after treatment with NKEVs (x axis), and normalized gene and protein counts (y axis), finding 868 and 152 significantly correlated transcripts and proteins, respectively (p< 0.05) (**Figures 3g, h**; **Supplementary File 7**). Several long non-coding RNA transcripts were significantly associated with cytotoxic ability (*lnc-MFHAS1, LINC01128, ITPKB-AS1, lnc-GCNT1*), as were transcripts coding for nucleic acid processing proteins (*CNBP, MBNL1, SNRPD2*). Protein coding transcripts and proteins involved in ubiquitination and proteasomal degradation pathways were positively correlated with cytotoxicity (*PSMD1, UBE2E1, FBXW11, MKRN1, PSMB7;* UBE2NL, PSMA4, PSMB2). Taken together, these data showed that patient NKEVs exhibit anti-tumor effects in experiments with both patient derived and cell line organoids, significantly lowering the dose of cisplatin required to elicit an effect. Furthermore, the extent of cytotoxicity is correlated with molecular EV protein and RNA cargo that may influence nucleic acid processing and expression, and protein degradation and stability.

### NKEVs enhance cytotoxic lymphocyte recruitment to the tumor organoid

**Figure 4:**
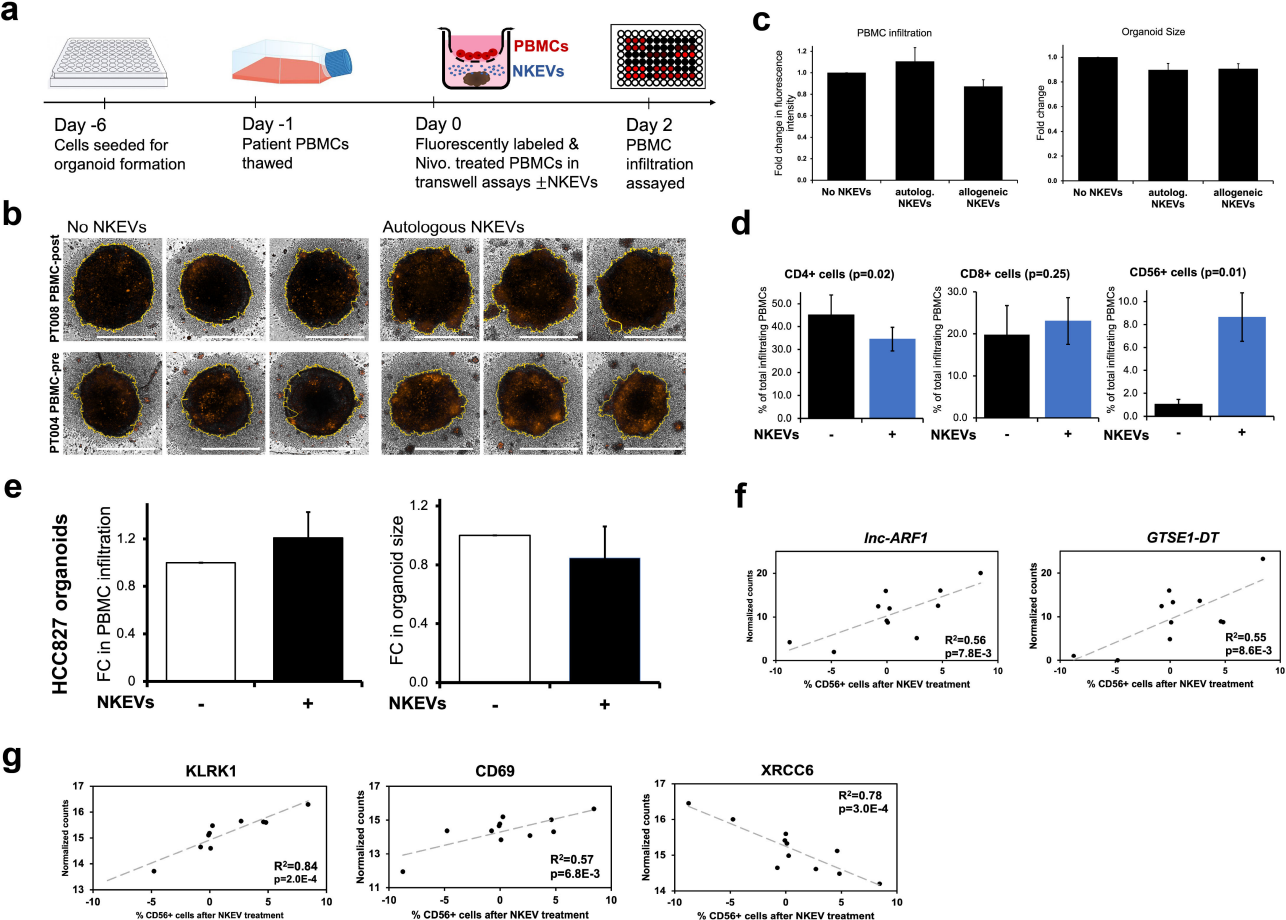
Patient NKEVs influence the immune cell population distribution in tumor organoid experiments. Fluorescently labeled, Nivolumab-treated patient PBMCs were placed in transwells above the organoids, with or without NKEVs, and immune infiltration was assayed 48 hours later **(a)**. Projected Z-Stack images of lung cancer organoids after infiltration, where the fluorescent signal is present, primarily around the organoid edges **(b)**. Morphometric analysis of transwell experiments with patient derived organoids found that in the presence of NKEV addition, PBMC infiltration increases slightly with autologous EVs but not significantly, and organoid size diminishes (likewise non-significantly) **(c)**. Flow cytometry analysis of the lymphocytes embedded in the organoids 48 hours later found a significant shift in the population of CD4+ and CD56+ cells after NKEV treatment **(d)**. Morphometric analysis of experiments with HCC827 organoids mirrors the findings from patient tumor organoids **(e)** Transcripts **(f)** and proteins **(g)** that correlate significantly with CD56+ cell recruitment are displayed as scatter plots with corresponding R^2 and p values. In **(b)** the yellow lines generated by the software for morphometric analysis trace the organoid boundaries, and horizontal white bars represent 500 um. Error bars in **(c-e)** are SEM. “autolog. NKEVs” denotes PBMCs, tumor organoid cells and NKEVs all derived from the same source, whereas “allogeneic NKEVs” refers to cross-testing NKEVs from a different donor **(c)**. Statistical analysis was done via t test **(d, e)** and one-way Anova **(c)**. Organoid experiments were done in 3–6 replicates per condition, depending on EV and organoid availability, and organoids were pooled for flow cytometry analysis.

To investigate the utility of NKEVs as a potential adjuvant to ICI therapy, experiments were set up with patient organoids and fluorescently labeled PBMCs treated with PD1 inhibitor Nivolumab, in transwell assays, either in the presence or absence of NKEVs (**Figure 4a**). After 48 hours, 3D Z-stack imaging was used to examine the extent of immune cell infiltration (**Figure 4b**). The addition of NKEVs, whether autologous or allogeneic, did not have a statistically significant effect on enhancing overall PBMC infiltration, as measured via fluorescence intensity; organoid size decreased slightly in the presence of EVs, but not significantly (**Figure 4c**; **Supplementary Figure 2a**). However, analyzing the identity of the infiltrating PBMCs via flow cytometry revealed NKEV driven changes. On average, 45% of organoid infiltrating cells were CD4+ (marking T helper cells), 20% CD8+ (cytotoxic T cells), and only 1% CD56+ (NK cells), although the exact numbers varied across patients, and generally reflected the initial circulating lymphocyte distribution (**Figure 4d**; **Supplementary Figure 2c**). Addition of NKEVs favorably shifted the T cell balance to fewer CD4+ cells (10% decrease, p=0.02), and slightly more CD8+ cells (not significant). Strikingly, the proportion of infiltrating CD56+ cells was significantly enhanced in the presence of NKEVs (7.6% increase, p=0.01) (**Supplementary File 8**). PT002 organoid and PBMC co-culture experiments, for example, only had measurable CD56+ infiltration post NKEV treatment (**Supplementary Figure 2c**). HCC827 cell line organoids assays showed a slight but non-significant increase in immune cell infiltration and diminished organoid size upon NKEV treatment (**Figure 4e**; **Supplementary Figures 2b, d**). The proportion of cytotoxic CD8+ and CD56+ cells was slightly elevated after NKEV addition, albeit not significantly. Linear regression analysis comparing the extent of recruitment and molecular EV cargo found 187 and 337 significantly correlated transcripts and proteins, respectively (p< 0.05). Like cytotoxicity, NKEV recruitment ability was correlated to long noncoding RNA transcript cargo (*lnc-ARF1, GTSE1-DT, lnc-SLFN12*) (**Figure 4f**; **Supplementary File 8**). Interestingly, there was a significant positive relationship between recruitment and the expression levels of stimulatory NK cell receptors KLRK1 (NKG2D) and CD69, and negative correlations between CD56+ cell recruitment and proteins associated with DNA repair machinery (XRCC6, XRCC5) (**Figure 4g**).

**Figure 5:**
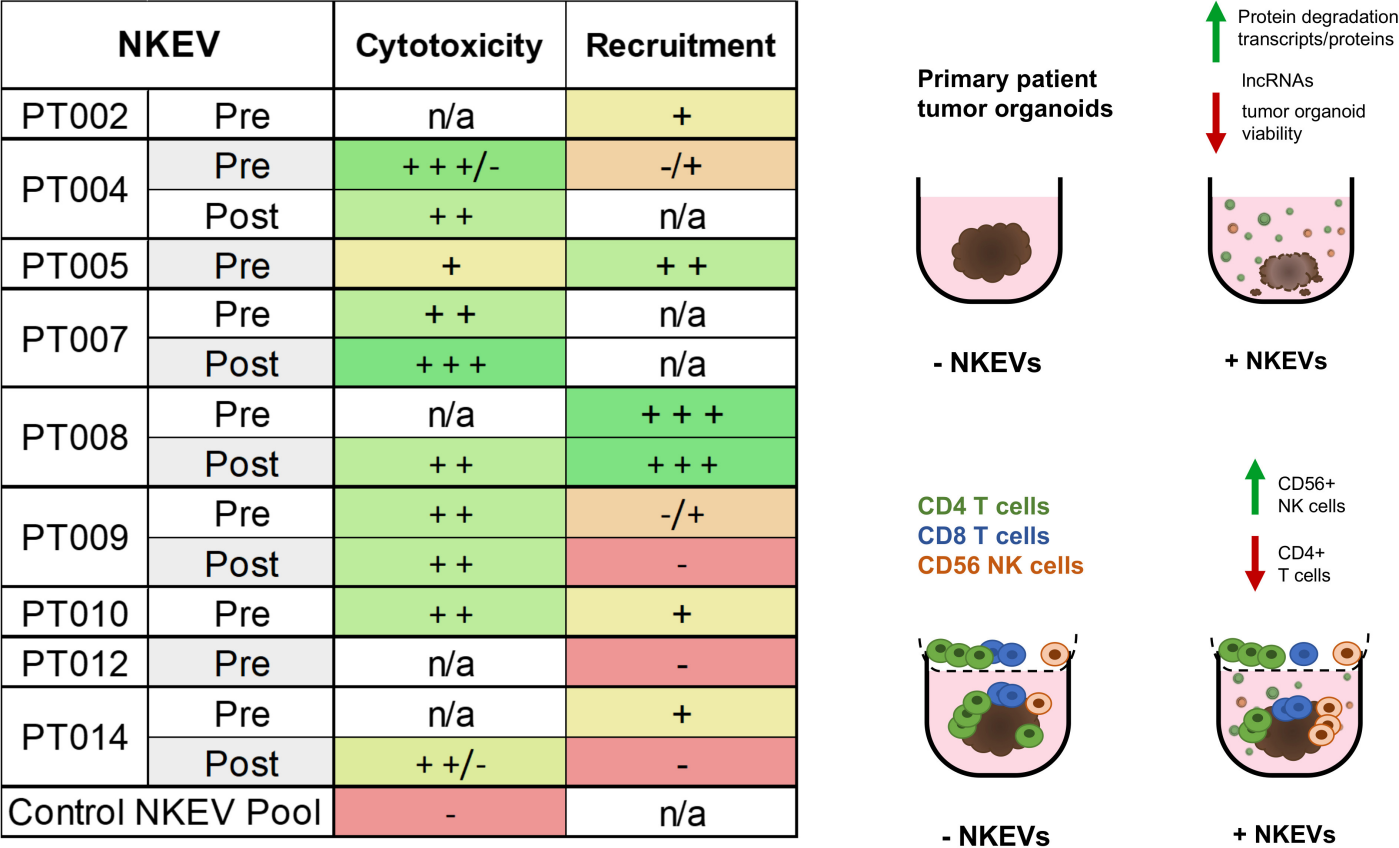
NKEVs often exhibit either enhanced cytotoxic functions, or CD56+ cell recruiting tendencies. A summary of the NKEV cytotoxicity and immune infiltration experiments with cell line organoids is presented as a table, while the visual diagram highlights the major findings from the EV cargo profiling.

Altogether, these data suggested NKEVs exhibit functional specialization linked to their cellular origin and molecular cargo, favoring the infiltration of cytotoxic immune cells into tumors. NKEV cytotoxicity in the absence of PBMCs was likewise linked to RNA and protein cargo and correlated with the ratio of immunomodulatory and cytotoxic NK parent cells (**Figure 5**).

## Discussion

While NSCLC patient outcomes have improved with the deployment of ICI therapeutics, many patients do not respond, have harmful side-effects, or develop resistance, necessitating novel therapeutic strategies to prolong life. Adoptive T cell strategies utilizing CARs and TCRs have been disappointing due to challenges overcoming the absence of stable tumor antigen expression and the need for HLA restriction. Therapies employing NK cells, which advantageously target tumor cells without MHC-dependent activation and are less likely than T cells to elicit severe side effects, are the topic of extensive research and dozens of clinical trials for both hematological and solid tumors, including lung cancer (15, 43). In our study, we profiled the immune cell population in a small cohort of NSCLC patients and explored the utility of NKEVs as a therapeutic augment in tumor organoid experiments.

In peripheral circulation, the distribution of each immune cell type varied between individuals, although all cell types were represented in every sample (**Figure 1**; **Supplementary Figure 1**; **Supplementary File 2**). Interestingly, there was a slight but significant drop in NK cells post-surgery, which ties in with current knowledge on the effects of tumor resection and choice of anesthetics potentially creating an immunosuppressive environment with increased levels of proinflammatory and proangiogenic factors (44–48). Comparing LUAD and LUSC participants found significant differences in genes involved in antigen processing and presentation, and cytokine signaling across various circulating PBMC populations, mirroring recent work hinting at similar lung cancer subtype specific interactions in the TME (49). Given that antigen processing and presentation is important for tumor recognition and ICI efficacy, as well as mechanisms of immune evasion and resistance, understanding the molecular differences between LUAD and LUSC patients is critical for informing treatment options (50–52).

Pivotal aspects of cell identity are defined by protein expression, localization and interactions, which are not captured by transcriptomic analysis. We used CITE-seq to quantitatively label cell surface proteins CD16 and CD56, which allowed us to identify NK subtypes, together with gene expression analysis. We established an NK_im_ cell cluster, characterized by upregulation of classic cytokine receptors and transcription factor genes (*IL2RB, IL7R, TCF7, KLRC1*), a large NK_ctx_ cluster expressing high levels of cytotoxic and effector protein coding genes (*GZMB/H, PRF1, FCGR3A*) and a small proliferating cluster marked by expression of MKI67, largely in agreement with previous NK scRNAseq work (50). No significant differences in NK subtype distribution were found when comparing pre- and post-resection or LUAD/LUSC samples. However, analyzing tumor grade and NK subtypes found a shift towards larger ratios of NK_im_/NK_ctx_ cells in patients with higher-grade tumors. Within the NK_im_ subcluster, gene expression of key receptors and cytokines (*Il15, CXCR3, KLRG1*) correlated significantly with tumor grade. Il15 bolsters NK cell activity and expansion, although prolonged exposure may lead to hyporesponsiveness (53, 54). Given that NK_im_ cells may be enriched in the tumor infiltrating immune milieu, and that levels of NK subsets can hold predictive value for therapy response, understanding how the population changes over the course of disease is useful (55).

There was no NK cell cluster present in the tumor infiltrating immune population, as analyzed by snRNAseq, and subclustering found predominantly CD4 T/Treg cells, with CD8 T cells constituting only 20-40% of infiltrating T cells (**Figure 1**; **Supplementary Figure 1**). These results are in line with previous findings of NK cell depletion at the TME, and a skew towards larger numbers of CD4 T cells, although exact percentages vary across study, and is likely a product of experimental design, methodology and sample type (56–58). Expanding the cohort to include more samples would improve resolution of cell types and states identified.

NKEVs have been the topic of extensive research over the past decade, but to our knowledge, no attempts to profile the entire NKEV protein and RNA cargo from primary patient derived cells have been made, and the effect of NKEV treatment on tumor infiltrating immune populations has not been explored. We took advantage of PBMCs from our NSCLC cohort to isolate NK cells via negative selection and expand them feeder-free, which eliminates potentially confounding effects from feeder cell derived EVs. As various culture conditions and expansion strategies can significantly influence NK cell identity and function, we confirmed the presence of CD56 and CD16 two weeks post isolation, using flow cytometry (**Supplementary File 3**) (53, 59, 60). However, comprehensive transcriptomic analysis of expanded NK cells was not performed; future work incorporating scRNAseq following prolonged *in vitro* culture could offer valuable insight into NK cell biology and its impact on secreted NKEV cargo.

To maintain EV membrane integrity and avoid aggregation or protein co-precipitation issues caused by other EV isolation methods, we captured vesicles with size exclusion chromatography and used whole transcriptome RNAseq and mass spectrometry to profile the EV cargo, identifying 13,450 genes (gene count >10) and 4358 proteins (**Figure 2**) (61, 62). DE analysis found few significant (p adj. <0.05) differences in RNA and protein cargo dependent on time point (pre- vs. post-surgery), or diagnosis (LUAD vs. LUSC), although the transcript encoding *ERAP2* was significantly enriched in LUAD EVs, suggesting some of the cancer subtype specific differences in antigen processing and presentation observed in circulation are maintained in culture, and reflected in EVs.

We singularized the surplus lung tumor tissue retrieved during surgery, when available, and generated 3D organoid/spheroid structures using previously established protocols (**Figure 3**) (63). Calculations for cisplatin IC50 dosage showed considerable variability between patient samples, with some being very sensitive (PT002, PT014) and others resistant (PT004), although a larger sample size would be necessary to investigate mechanisms of resistance and correlate them to various biological factors. However, it does underscore the clinical utility of primary patient tissue for testing personalized treatments and studying disease, as establishing organoids takes only 2–3 weeks. Treating the patient tumor organoids with patient derived NKEVs resulted in significant cytotoxicity. Specifically, NKEV addition led to 2-3x higher organoid viability loss than cisplatin alone. In a clinical setting, reducing drug concentrations can improve patient compliance, lessen side effects, and decrease the selective pressure for evolving mechanisms of resistance (64). Thus, developing auxiliary agents that enable dose reduction of the primary drug is highly beneficial. No significant differences in EV efficacy based on time point (pre/post) or experimental combination (autologous vs allogeneic EVs) were observed. Cancers are often associated with impaired cytotoxicity and function in the NK cell population (19, 65). In our hands, patient NKEVs were more effective than control NKEVs, suggesting the dysfunction does not extend to NKEVs isolated from *ex vivo* expanded NK cells. Examining NKEV proteomics and transcriptomics normalized counts shows hundreds of proteins and genes exclusively found in cancer patient samples but not in the control pool, although levels of cytotoxic molecules (Perforin, Granzymes, Gnly) were not strikingly different between control and patient NKEVs (**Supplementary File 4**). However, these findings should be interpreted with caution due to their preliminary nature and limited sample size. Further experiments with a larger group of control samples could confirm these observations and identify the specific aspects of patient NKEVs that underlie their increased cytotoxicity. Interestingly, experiments with cell line organoids found a positive correlation between the extent of NKEV cytotoxicity and the starting ratio of the original NK_ctx_/NK_im_ cell population. While not entirely surprising, these findings illustrate that the NK cell subtype differences may be, in some capacity, reflected in their EV cargo, and should be considered when exploring NKEV centric therapeutics. Several long non-coding RNA transcripts were significantly correlated with anti-tumor activity, pointing to a previously unappreciated role for lncRNA in NKEV function. *LINC01128*, for example, which has been explored in several cancers and is thought to modulate miRNA expression, was negatively associated with NKEV cytotoxicity (66, 67). Several proteins and transcripts involved in protein degradation and ubiquitination, as well as RNA processing, were positively correlated with cytotoxicity. Future experiments carried out on additional immortalized NSCLC cell lines with diverse genetic backgrounds could validate and contextualize these results.

Not surprisingly, the presence of tumor infiltrating lymphocytes (TILs) with cytotoxic properties is associated with improvements in prognosis and response to treatment in various cancers (68, 69). For NSCLC, the addition of immune checkpoint inhibitor Nivolumab, which targets PD-1, and subsequently significantly improved outcomes without increasing adverse effects (70). We investigated whether NKEVs, as a potential adjuvant to ICI therapy, can favorably impact tumor immune infiltration. To this end, we fluorescently labeled patient PBMCs, treated them with Nivolumab, and explored infiltration into the patient derived organoids in transwell assays, as previously described (**Figure 4**) (63). Assessment of infiltrating lymphocytes showed a large percentage of CD4+ cells and little to no CD56+ cells, reminiscent of the infiltrating population found in the tumor samples, where snRNAseq analysis found CD4 T cells were more abundant than cytotoxic CD8 T cells, and NK cells were not detected. NKEV treatment resulted in slightly increased overall infiltration, and diminished organoid size, although not significantly. The proportion of infiltrating cells changed favorably with NKEV addition, to fewer CD4+ cells and significantly more CD56+ cells. Examining the EV cargo in cell line organoid experiments found that expression of NKG2D and CD69 proteins was favorably associated with the extent of CD56+ cell infiltration. Activating receptor NKG2D plays well documented roles in NK cells’ effector function and ability to co stimulate cytotoxic T cells, and previous work has found that NKG2D ligand inhibition impairs the NKEVs’ antitumor activity, in experiments with NK-92 EVs (26, 71). The improvement in CD56+ cell infiltration was striking, with implications for therapies aimed at increasing TILs. However, additional mechanism focused research is required to distinguish the direct effects of NKEVs on tumor organoids from their impact on immune cells, and understand how NKEVs modulate immune cell activation, cross talk, and migration into the tumor organoid. Further exploration of NKEV-driven immune infiltration alone/in combination with ICIs or other standard NSCLC treatments will also help determine the optimal context for NKEV based therapeutics.

Together, the NKEV experiments demonstrated that cancer patient-derived NKEVs exert greater cytotoxic effects than control NKEVs, significantly reducing the cisplatin dose required for anti-tumor activity and, in PBMC co-culture assays, can enhance the recruitment of CD56+ cells. Interestingly, some of the top performing NKEVs in the immune recruitment experiments tended to perform worse in cytotoxicity experiments and vice versa, suggesting that NKEVs might contain subtypes with different cytotoxic and immune signaling abilities, potentially with a tendency to favor one function over the other (**Figure 5**).

Harnessing NK cells and the EVs they produce for therapy is attractive due to their innate effector functionality. We leveraged the primary human tissues and cells from our NSCLC cohort, and employed a range of innovative methods like single cell CITE-seq, low-input EV cargo multi-omics analysis to conduct an investigation into the immune cell biology of lung cancer and explore NKEVs as a potent therapeutic strategy. However, we acknowledge 1) the small sample size of our cohort and 2) the *in vitro* nature of the experiments, which limits both statistical power and the ability to generalize these findings to *in vivo* tumor biology. Validation in a larger cohort with diverse lung cancer subtypes and mutation backgrounds would address these challenges. It would enable investigation into how common NSCLC mutations or immune populations influence response to NKEV treatment, and whether the results are broadly applicable. Incorporation of animal model work could strengthen the biological relevance of our findings. Furthermore, our results raise mechanistic questions about how NKEV’s exert their cytotoxic functions. Previous research with various animal models, primary and immortalized NK cells have described NKEV cargo (*mir-186*, FasL, Perforin-1, granzymes, cytokines) with antitumor properties, and found they can augment NK cell proliferation and T cell activation. Unlike their cell of origin, NKEVs are not affected by the immunosuppressive environment of the TME (27, 29, 72–76). The horizontal transfer of EV cargo from immune EVs to cancer cells has indeed been observed in various contexts (77, 78). While the experiments described here provide very limited mechanistic insight, the multi-omics analysis of NKEV cargo offers potential clues. In cell line organoid experiments, expression levels of both coding and noncoding transcripts significantly correlated with cytotoxicity, suggesting possible lateral transfer of genetic material to target cells. Protein levels of receptor KLRK1 (aka NKG2D), which binds stress-induced ligands on recipient cells and results in activation, was positively associated with NK cell infiltration, which hints at receptor-ligand interactions potentially underpinning the NKEV’s immune recruiting abilities. Another aspect of EV biology that warrants further exploration is the diversity in secreted vesicle subtypes- including exosomes, microvesicles, apoptotic bodies, exomeres- each with potentially distinct biogenesis, structure, size and cargo. Indeed, nanotracking analysis of patient derived NKEVs shows a distribution of particle diameter which may reflect distinctly sized populations. We did not attempt to separate and study the EV subpopulations individually in our study, choosing instead to treat the group in bulk, but understanding the heterogeneity in the NKEV milieu distribution and cargo, and determining the extent to which each subpopulation exhibits anti-tumor activity will be helpful for establishing NKEV based therapeutics.

Overall, our results confirmed the scarcity of cytotoxic T and NK cells at the tumor site, identified cancer subtype specific differences in circulating immune cells, and showed that patient derived NKEVs are uniquely cytotoxic and enhance tumor infiltration of NK cells in organoid experiments. Together, our findings demonstrate the potential for exploiting NKEVs as a novel adoptive therapeutic for NSCLC.

## Data Availability

The datasets presented in this study can be found in online repositories. The names of the repository/repositories and accession number(s) can be found below: https://www.ncbi.nlm.nih.gov/geo/, GSE274588 https://www.ncbi.nlm.nih.gov/geo/, GSE274595 https://www.ncbi.nlm.nih.gov/geo/, GSE274584.

## Supplementary material

Supplementary Figure 1 Profiling circulating and tumor infiltrating immune cells in a cohort of 10 NSCLC patients. Cell Typist was used to assess cell identity, and an overview of the topmost highly expressed genes in each cluster are displayed as dot plots for PBMCs (n=19) **(a)** and tumor cells (n=8) **(h)**. The overall distribution of PBMCs after analysis and quality filtration in UMAP format was shown for samples grouped by time point (left) and by patient (right) **(b)**. UMAPS for antibody labeling experiments (n=19) found CD16 expression primarily in NK cells and non-classical monocytes and high CD56 expression confined to a small subset of NK cells. The isotype control antibody had slight non-specific labeling, particularly within the classical monocyte cluster **(c)**. A diagram of the NK cell subclustering shown as a UMAP and bar graph found all patients were represented in the 3 subtypes and expression of MKI67 is confined to the proliferating subcluster **(d)**. Expression of representative normalized gene counts for the NK immunomodulatory subcluster **(e)** and NK cytotoxic subcluster **(f)** are shown grouped by pathological tumor grade. Clusters of cells belonging to the tumor proper were filtered out of the analysis based on expression of tumor specific markers **(g)**.

Supplementary Figure 2 Patient NKEVs influence the immune cell population distribution in tumor organoid experiments. The morphometric analysis from immune cell infiltration experiments with patient NKEVs and either patient derived organoids **(a)** or the lung cancer cell line HCC827 organoids **(b)** is shown as bar plots for fluorescence intensity and organoid size. The infiltrating population of CD4+, CD8+, and CD56+ cells in the patient organoid experiments is displayed as bar graphs, along with the percentage of starting CD4 T cells, CD8 T cells, and CD56 NK cells in the PMBCs used, as assayed by scRNAseq **(c)**. The infiltrating population of CD4+, CD8+, and CD56+ cells in the cell line organoid experiments is also shown in bar graph form **(d)**. Representative gating strategy for flow cytometry analysis is outlined, where dead cells were excluded via Zombie Violet viability staining, immune cells were identified by DiI staining, and CD4+, CD8+ and CD56+ were counted out of the live, DiI population.
