# Supplementary figures and images for: NK cell-derived extracellular vesicles enhance cytotoxicity and immune cell recruitment in non-small cell lung cancer
Source: Front Immunol. 2025 Jul 24;16:1633010. doi: 10.3389/fimmu.2025.1633010 (PMC12355224; doi:10.3389/fimmu.2025.1633010)

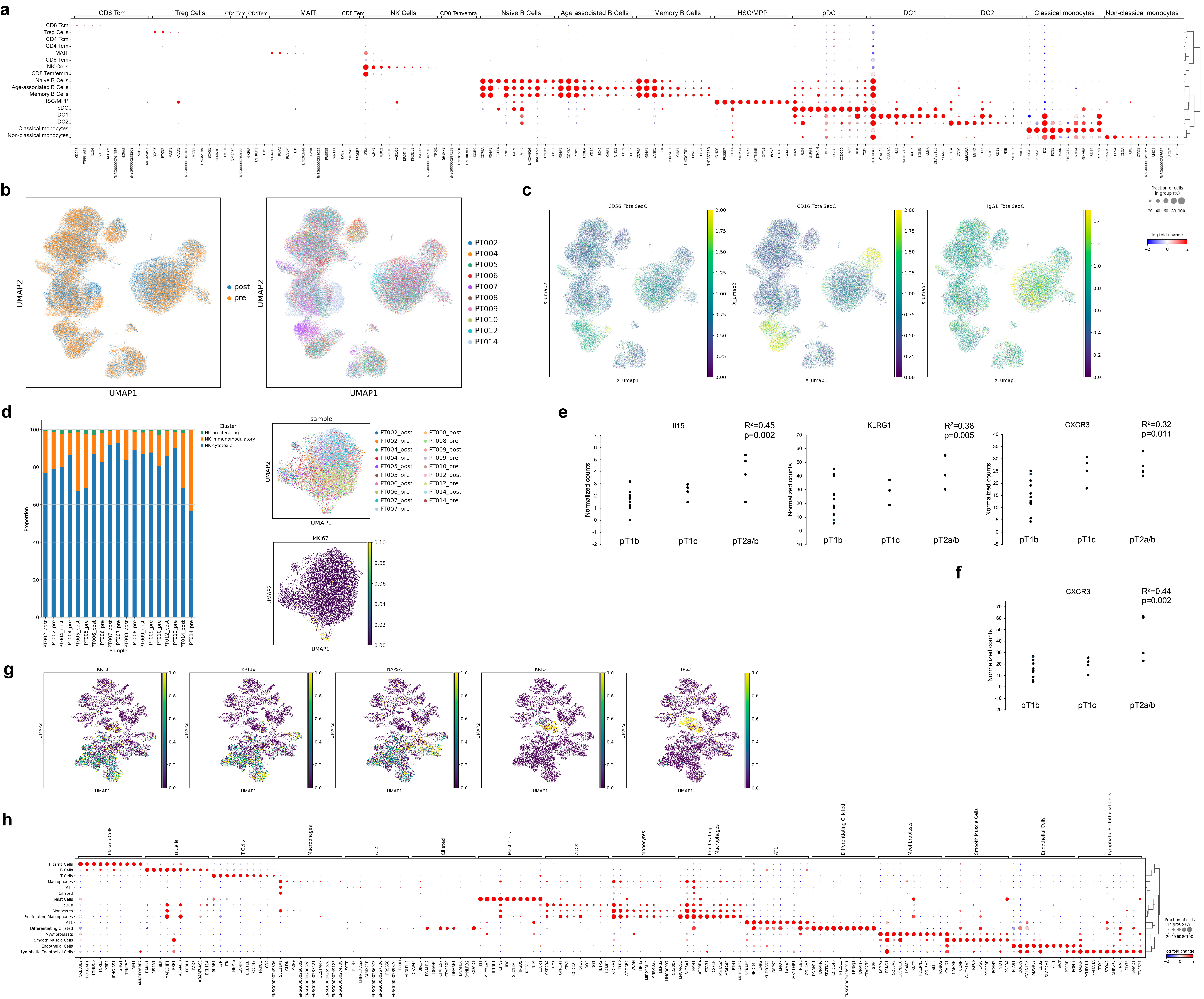

Supplement: Supplementary Figure 1 — Profiling circulating and tumor infiltrating immune cells in a cohort of 10 NSCLC patients. Cell Typist was used to assess cell identity, and an overview of the topmost highly expressed genes in each cluster are displayed as dot plots for PBMCs (n=19) (a) and tumor cells (n=8) (h). The overall distribution of PBMCs after analysis and quality filtration in UMAP format was shown for samples grouped by time point (left) and by patient (right) (b). UMAPS for antibody labeling experiments (n=19) found CD16 expression primarily in NK cells and non-classical monocytes and high CD56 expression confined to a small subset of NK cells. The isotype control antibody had slight non-specific labeling, particularly within the classical monocyte cluster (c). A diagram of the NK cell subclustering shown as a UMAP and bar graph found all patients were represented in the 3 subtypes and expression of MKI67 is confined to the proliferating subcluster (d). Expression of representative normalized gene counts for the NK immunomodulatory subcluster (e) and NK cytotoxic subcluster (f) are shown grouped by pathological tumor grade. Clusters of cells belonging to the tumor proper were filtered out of the analysis based on expression of tumor specific markers (g). [file Image1.jpg]

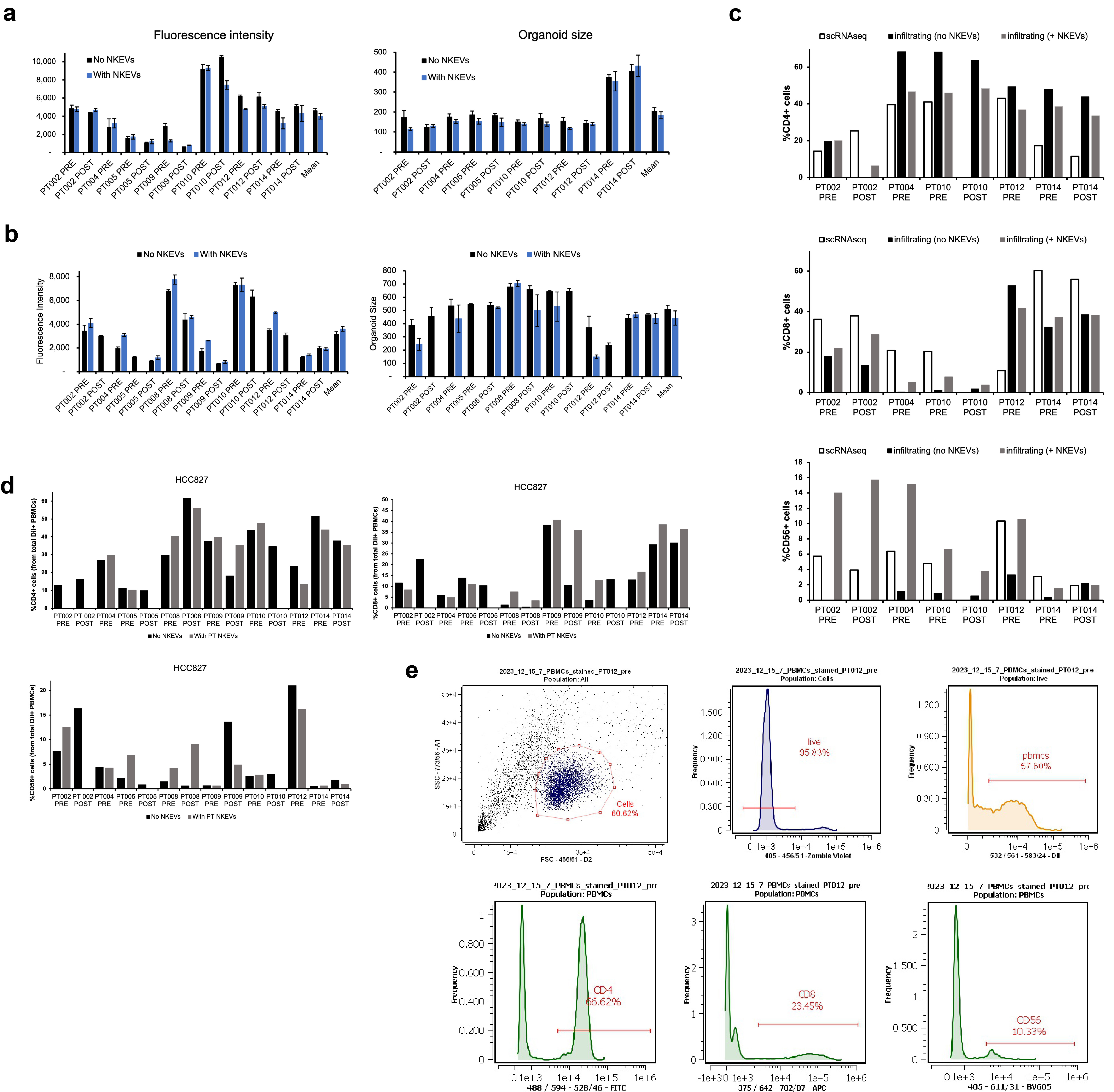

Supplement: Supplementary Figure 2 — Patient NKEVs influence the immune cell population distribution in tumor organoid experiments. The morphometric analysis from immune cell infiltration experiments with patient NKEVs and either patient derived organoids (a) or the lung cancer cell line HCC827 organoids (b) is shown as bar plots for fluorescence intensity and organoid size. The infiltrating population of CD4+, CD8+, and CD56+ cells in the patient organoid experiments is displayed as bar graphs, along with the percentage of starting CD4 T cells, CD8 T cells, and CD56 NK cells in the PMBCs used, as assayed by scRNAseq (c). The infiltrating population of CD4+, CD8+, and CD56+ cells in the cell line organoid experiments is also shown in bar graph form (d). Representative gating strategy for flow cytometry analysis is outlined, where dead cells were excluded via Zombie Violet viability staining, immune cells were identified by DiI staining, and CD4+, CD8+ and CD56+ were counted out of the live, DiI population. [file Image2.jpg]
